# Protective Effect of Bioactivity Guided Fractions of *Ziziphus jujuba* Mill. Root Bark against Hepatic Injury and Chronic Inflammation via Inhibiting Inflammatory Markers and Oxidative Stress

**DOI:** 10.3389/fphar.2016.00298

**Published:** 2016-09-07

**Authors:** Raghuram Kandimalla, Suvakanta Dash, Sanjeeb Kalita, Bhaswati Choudhury, Sandeep Malampati, Kasturi Kalita, Bhupalee Kalita, Rajlakshmi Devi, Jibon Kotoky

**Affiliations:** ^1^Drug Discovery Laboratory, Institute of Advanced Study in Science and TechnologyGuwahati, India; ^2^Girijananda Chowdhury Institute of Pharmaceutical ScienceGuwahati, India; ^3^School of Chinese Medicine, Hong Kong Baptist UniversityHong Kong, China; ^4^Department of Pathology, Hayat HospitalGuwahati, India; ^5^Jawaharlal Nehru Centre for Advanced Scientific ResearchBengaluru, India

**Keywords:** liver toxicity, antioxidant, cytokines, inflammation, oxidative stress

## Abstract

The tribal communities of North Eastern India rely on herbal medicine to cure various disease conditions. *Ziziphus jujuba* Mill. (*Rhamnaceae*) is one of such medicinal plants used for curing liver ailments, insomnia, anemia, diarrhea, diabetic complications, cancer, and loss of appetite. The present study was aimed to describe the protective ability of *Z. jujuba* root bark (ZJRB) against hepatic injury and chronic inflammation. Bioactivity guided fractionation of *Z. jujuba* methanol extract (ZJME) was performed using different solvents of increasing polarity viz. hexane (ZJHF), chloroform (ZJCF), ethyl acetate (ZJEAF), water (ZJWF), and residue (ZJMR). *In vitro* antioxidant results revealed that both ZJME and ZJWF possess strong antioxidant activity among all the fractions and mother extract tested. Further, ZJME and ZJWF showed significant protection against CCl_4_ intoxicated HepG2 cell lines by means of increased cell viability and decreased LDH levels compared to control group. ZJME at 200, 400 mg/kg and ZJWF at 50, 100 mg/kg inhibited the lipid peroxidation and significantly restored the liver function markers (AST, ALT, ALP, LDH, SOD, and CAT) and cytokine levels (TNF-α, Il-1β, and Il-10) in CCl_4_ induced acute liver damage in rats. All the results were comparable with standard drug silymarin which was further confirmed by histopathology analysis of liver. Similarly, inflammation and increase inflammatory cytokines levels of carrageenan induced paw edema in rats have been refurbished to normal levels on par with the standard drug indomethacin. ZJWF demonstrated potent response than ZJME in all the biological tests conducted. The results of the study signify the ability of ZJRB as good therapeutic agent for liver toxicity and chronic inflammation.

## Introduction

During the metabolism process, numerous number of reacting oxygen species (ROS) like superoxide anions, hydroxy radicals and hydrogen peroxide generates inside the body. This ROS covalently binds to the different cell organelles and involves in causing diseases like cancer, inflammation, arthritis, aging, and cardiac problems (Chen et al., 2011). These reactive intermediates mainly involve in membrane lipid peroxidation which causes genesis of non-alcoholic steatohepatitis and liver carcinoma (Ha et al., 2010). The liver is the complex chemical factory and main organ of the human body which involves in vital functions like cleansing blood, vitamin synthesis, regulation of the supply of body fuel, cholesterol regulation, balancing hormone regulation, and drug metabolism. Different ailments like the virus, chronic alcoholism, and toxic chemicals cause liver damage and failure which is a major problem worldwide (Hou et al., 2013). ROS plays an important role in hepatic fibrogenesis through platelet deriving growth factor. In extreme conditions, ROS activates oxidative stress mediated inflammation and leads to hepatocellular carcinoma (Muriel, 2009).

Inflammation is the defense mechanism by an organism against pathogens, irritants or physical injury, which also implicated in many diseases like multiple sclerosis, rheumatoid arthritis, complications of sepsis, and ischemia-reperfusion injury. All these diseases associated with increased production of ROS due to oxidative stress. Inflammation stimuli trigger the macrophages to produce and release of inflammatory cytokines like tumor necrosis factor alpha (TNF-α) and interleukin 1β (IL-1β) which causes chronic inflammation and pain (Huang et al., 2012; Salzano et al., 2014). There are a few treatment options available in the market for treating the liver ailments and inflammation with undesirable side effects, which demands the new treatment strategies.

Ayurvedic medicine has gained worldwide attention because of its effectiveness without causing unwanted side effects. Presently, the research community is focusing on thoroughgoing pharmacological validation and identification of biomarkers for the treatment of various disease conditions (Feng et al., 2011; Pareek et al., 2013; Choudhury B. et al., 2016). North Eastern India is a part of Indo-Burma biodiversity hotspot known for widespread availability of herbal medicine. *Ziziphus jujuba* Mill. (Rhamnaceae) is one such plant used by traditional healers for the treatment of liver ailments, cancer, diabetic complications, insomnia, anemia, diarrhea, and loss of appetite (Elaloui et al., 2016). This plant possesses different chemical components like alkaloids, flavonoids, glycosides, and phenolic group of compounds (Yoshikawa et al., 1997; Pawlowska et al., 2000). *Z. jujuba* was reported for anti-cancer (Huang et al., 2007), anxiolytic (Peng et al., 2000), anti-complementary (Lee et al., 2004), and hypoglycemic (Iganacimuthu and Amalraj, 1998) activities. Different parts of this plant have been using by tribal communities of North East India for the treatment of different disease conditions. Especially root bark of this plant has been using for the treatment of liver toxicity, jaundice, and diabetic complications. These claims were authenticated by Dr. Dinesh Boruah, Senior scientist at North East Indian Ayurvedic Research Institute, Govt. of India, Guwahati, Assam. Since there is no scientific validation of this folklore claim, the present study was aimed to scientifically validate the bioactive guided fractions of *Z. jujuba* root bark (ZJRB) against liver toxicity and chronic inflammation using *in vitro* and *in vivo* methods.

## Materials and Methods

### Chemical and Drugs

2,2-diphenyl-1-picryl hydrazyl (DPPH), 3-(4,5-dimethyl thiazol-2-yl)-5-diphe-nyl tetrazolium bromide (MTT), trypan blue, trypsin, carrageenan were obtained from Sigma-Aldrich Co, St Louis, MO, USA. Fetal Bovine serum (FBS) and Dulbecco’s Modified Eagle medium (DMEM) low glucose were purchased from Invitrogen, Life technologies, USA. Trichloroacetic acid (TCA), thiobarbituric acid (TBA), and ethylenediaminetetraacetic acid (EDTA) were obtained from Hi-Media Lab, Pvt. Ltd, Mumbai. TNF-α, IL-1β, & IL-10 kits were purchased from R&D systems, USA. Diagnostic kits for serum aspartate aminotransferase (AST), alanine aminotransferase (ALT), alkaline phosphatase (ALP), and lactate dehydrogenase (LDH) were purchased from Accurex Biomedical Pvt. Ltd, Mumbai. Superoxide dismutase (SOD) and catalase (CAT) assay kits were procured from Cayman, USA. All the other chemicals used in this study were of analytical grade and obtained from either Sigma-Aldrich or Merck.

### Plant Collection and Identification

*Ziziphus jujuba* plant root bark was collected from Guwahati, Assam (26.1833° N, 91.7333° E) in the month of May 2015 and identified by a taxonomist at North East India Ayurvedic Institute, Guwahati. A voucher specimen number (727/IASST/ 2014) was deposited at herbarium, Drug discovery laboratory, Institute of Advanced Study in Science and Technology (IASST), Guwahati, Assam for future reference.

### Extraction and Bioactivity Guided Fractionation

**FIGURE 1:**
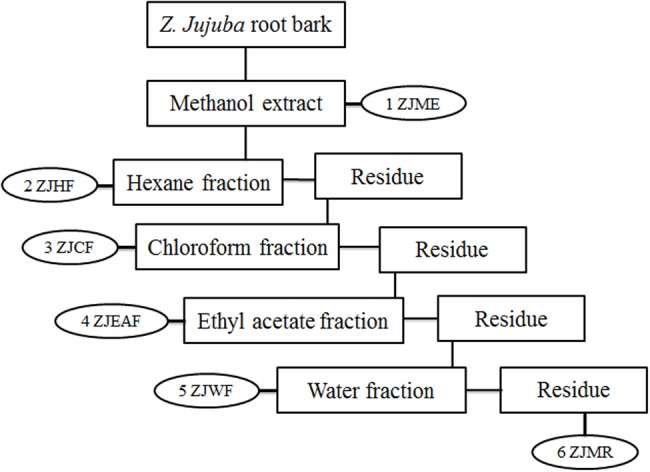
**Schematic representation of bioactive guided fractionation of *Z. jujuba* root bark.** ZJME, *Z. jujuba* root bark methanol extract; ZJHF, *Z. jujuba* root bark hexane fraction; ZJCF, *Z. jujuba* root bark chloroform fraction; ZJEAF, *Z. jujuba* root bark ethyl acetate fraction; ZJWF, *Z. jujuba* root bark water fraction; ZJMR, *Z. jujuba* root bark methanol residue.

*Ziziphus jujuba* root bark was shade dried at room temperature (25–27°C) and ground into coarse powder. About 10 kg of the dried powder was subjected to maceration with methanol for 72 h. The *Z. jujuba* root bark *methanol* extract (ZJME) was concentrated under pressure using rota evaporator (Buchi, Switzerland) to yield the dry residue. A part of ZJME (400 g) was subjected to gradual fractionation using different solvents of increasing polarity viz, hexane (ZJHF), chloroform (ZJCF), ethyl acetate (ZJEAF), and water (ZJWF). Briefly, 400 g of ZJME was suspended in 1 l of hexane and stirred vigorously by using magnetic stirrer for 24 h at room temperature. Further, the hexane fraction was collected by filtration and the undissolved residue was collected and dried to continue the further fractionation. After extracting with all the solvents, the remaining methanol residue was collected and dried (ZJMR). The schematic representation of bioactivity guided fractionation process is shown in **Figure 1**. Main mother methanol extract and its five fractions were prepared for testing biological activity and stored at 4°C. All the samples were tested within 3 months of extraction and thawed before use (Kandimalla et al., 2016a).

### *In vitro* Antioxidant Activity

#### DPPH Radical Scavenging Assay

The radical scavenging activity was evaluated by DPPH assay (Jingsheng et al., 2012). Briefly, 2.7 mL of 0.2 mM DPPH was added to 0.3 mL of the extract solution at various concentrations. The mixture was shaken vigorously and incubated at room temperature for 1 h before the absorbance was measured at 517 nm. The radical scavenging activity was calculated as follows: scavenging rate = [(*A*s - *A*i)/*A*s] × 100, where *A*s is the absorbance of pure DPPH and *A*i is the absorbance of DPPH in the presence of various extracts. Ascorbic acid at different concentrations identical to the experimental samples was used as references.

#### Reducing Power Assay

The reducing power of the extracts was estimated using the method by Choudhury B. et al. (2016). Increased concentrations of 0.2 mL extracts were mixed with 2.5 mL of phosphate buffer (0.2 M, pH 6.6) and 2.5 mL of potassium ferricyanide (1%). After incubation at 50°C for 20 min, 2.5 mL of TCA (10%) was added, and each mixture was centrifuged at 1000 rpm for 10 min. Then, 2.5 mL of the supernatant was collected and mixed with 2.5 mL of deionized water and 0.5 mL of ferric chloride (0.1%). The absorbance was measured at 700 nm. The increased absorbance of the reaction mixture indicated increased reducing power. BHT was used as standards for comparison.

#### Total Antioxidant Activity

Measurement of total antioxidant capacity of plant extracts was done by photochemiluminescence method in the Photochem instrument, Germany. The lipophilic and hydrophilic antioxidants were measured with the commercially available kits from by Photochem (Germany) according to the protocol given by the manufacturer. Total antioxidant activity of plant extracts was expressed in trolox equivalents for lipophilic antioxidants and ascorbic acid equivalents for hydrophilic antioxidants (Kalita H. et al., 2016).

#### Cell Culture

Human liver hepatoma cells (HepG2) were procured from National Center for Cell Sciences (NCCS), Pune, India. The cells were seeded (1 × 10^5^ cells/T_25_ flask) and cultured in DMEM with low glucose containing 10% FBS and Penstrap (antibiotic solution) in CO_2_ Incubator at 37°C. Stock culture was grown in 25 cm^2^ culture flasks and all experiments were performed in 96 well plates (Life Technologies, USA).

### *In vitro* Cytotoxicity Assay

Tetrazolium salt assay (MTT) was used to determine the cytotoxic concentration of ZJME and ZJWF (Kalita S. et al., 2015, 2016; Kandimalla et al., 2016b; Konwar et al., 2016). Briefly, the cells from the culture flask were trypsinized and seeded in 96-well plate at 5 × 10^3^ cells/well in a culture medium and incubated in the CO_2_ incubator at 37°C for 24 h. Then the culture medium from the wells was replaced with new media and ZJME and ZJWF at different concentrations (5, 25, 50,125, 250, and 500 μg/mL). After 72 h of incubation in the CO_2_ incubator at 37°C, media was removed and 20 μl of 4 mg/mL MTT (pH 7.4) was added to each well. The plate was incubated for four more hours and then the supernatant was removed following 100 μl of DMSO was added to each well and further incubated for 30 min to dissolve the formed formazan. Absorbance was read in at 570 nm by using microplate reader (Thermo Scientific, USA). The percentage growth inhibition was calculated by using the following formula-

$$\%Growth\,\;Inhibition=100-\left[\frac{Mean\,\;OD\,\;of\,\;individual\,\;test\,\;group}{Mean\,\;OD\,\;of\,\;Control\,\;group}\right]\times 100$$

#### Protective Effect of *Z. jujuba* Active Fractions on CCl_4_ Induced Toxicity in HepG2 Cell Lines

HepG2 cells at 1 × 10^5^ cells/mL were adjusted with DMEM medium containing 10% FBS. To each well of 96 well microtitre plate, 0.1 mL of diluted cell suspension was added and incubated at 37°C for 24 h in the CO_2_ incubator. After 24 h of incubation, the media was discarded and expose the cells with different drug treatments as follows-

**Group-I (Control):**
*Normal control:* Cells were treated with 100 μl of serum free culture medium for 24 h.
*DMSO control:* Cells were treated with 100 μl of serum free culture medium containing DMSO (0.25% v/v) for 24 h.
*Silymarin Control*: Cells were treated with 100 μl of serum free culture medium containing silymarin (200 μg/mL) for 24 h.
*ZJME Control:* Cells were treated with 100 μl of serum free culture medium containing ZJME (200 μg/mL) for 24 h.
ZJWF control: Cells were treated with 100 μl of serum free culture medium containing ZJWF (200 μg/mL) for 24 h.
**Group-II (CCl_4_ treatment):** Cells were treated with 100 μl of serum free culture medium containing 1.0% (v/v) CCl_4_ for 24 h.
**Group-III (Standard treatment):** Cells were treated with 100 μl of serum free culture medium containing 1.0% (v/v) CCl_4_ and silymarin at different concentrations (50, 100, or 200 μg/mL) for 24 h.
**Group-IV (ZJME treatment):** Cells were treated with 100 μl of serum free culture medium containing 1.0% (v/v) CCl_4_ and ZJME at a concentration of (50, 100, or 200 μg/mL) for 24 h.
**Group-V (ZJWF treatment):** Cells were treated with 100 μl of serum free culture medium containing 1.0% (v/v) CCl_4_ and ZJWF at a concentration of (50, 100, or 200 μg/mL) for 24 h.

#### Cell Viability

Trypan blue exclusion assay (Rambabu and Vijayakumar, 2014) was performed to determine the cell viability. Briefly, after the exposure of cells to different treatments, cells from the wells were trypsinized and centrifuged at 1000 × *g* for 10 min at 4°C. The pellet was resuspended in 1 mL phosphate buffer saline (PBS), and then 0.1 mL of cell suspension was mixed with 0.1 mL of 0.2% trypan blue. Cells were counted using hemocytometer under light microscope and the percentage of viable cells was determined by using the following formula:

$$\%Viability=\frac{No\,\;of\,\;unstained\,\;cells}{Total\,\;cell\,\;count}\times 100$$

#### Measurement of Lactate Dehydrogenase (LDH)

After completion of the drug treatment period, culture media from the treatment groups were centrifuged at 2000 rpm for 15 min to separate the supernatant. LDH levels in the supernatant were measured by using Ecoline diagnostic kit as per the instructions are given by the manufacturer. The amount of enzyme catalyzes the conversion of lactate to pyruvate to produce 1.0 μmol of Nicotinamide adenine dinucleotide (NADH) per minute describe as one unit of LDH activity.

#### Acute Toxicity Studies

Acute oral toxicity studies were performed according to Organization for economic cooperation and development 423 (OECD 423) guidelines to test chemicals. Swiss albino mice of either sex (*n* = 6) were randomly selected for the study. Animals were kept overnight fasting with free access to water but not food, next day morning single dose of ZJME and ZJWF 2000 mg/kg body weight were administered orally to three animals of each group. Behavioral changes were observed for 24 h after the drug administration and animals were observed for 14 days, if mortality was observed in two out of three animals, then the dose was identified as the toxic dose. If mortality was observed in one animal, experiment was repeated again with the same dose to confirm the toxic dose. On further observation of mortality, the experiment was continued with low doses (300, 50, and 5 mg/kg body weight; Kandimalla et al., 2016d).

### Effect of *Z. jujuba* Active Fraction in CCl_4_ Intoxicated Rats

#### Animals

Adult male Wistar rats weighing 150–200 g were obtained from the Institute of Advanced Study in Science and Technology (IASST), Guwahati (India). Animals were housed and maintained at 24°C ± 1°C, relative humidity of 45–55% and 12:12 h dark/light cycle. Animals were free to access the water and standard pellet diet (Provimi Animal Nutrition Pvt. Ltd., India) throughout the experiment and all experiments were carried out between 09:00 and 17:00 h. The experimental procedures were approved by the Institutional Animal Ethics Committee (IAEC) of IASST, Guwahati (IASST/IAEC/2014-15/746) and all the experiments conducted in accordance with the CPCSEA (Committee for Purpose of Control and Supervision of Experimentation on Animals) guidelines.

#### Experimental Design and Drug Treatment

Adult male Wistar rats, a total of 42 animals were randomly divided into seven groups of six animals in each group. All the drug treatment was continued for 14 days and on 14th day single dose of CCl_4_ 1.5 mg/kg, in 1:1 dilution with olive oil was given in i.p. route.

**Group I:** D.W for 14 days orally + CCl_4_ 1.5 mg/kg on 14th day.
**Group II:** 0.3% CMC for 14 days orally + CCl_4_ 1.5 mg/kg on 14th day.
**Group III:** Silymarin 100 mg/kg in 0.3% CMC for 14 days orally + CCl_4_ 1.5 mg/kg on 14th day.
**Groups IV and V:** ZJME at 200 and 400 mg/kg in 0.3% CMC for 14 days orally + CCl_4_ 1.5 mg/kg on 14th day.
**Groups VI and VII:** ZJWF at 50 and 100 mg/kg in 0.3% CMC for 14 days orally + CCl_4_ 1.5 mg/kg on 14th day.

After 48 h of CCl_4_ administration, all the animals were sacrificed to collect blood and liver for biochemical estimation and histopathology analysis.

#### Serum Biochemical Estimation

Blood was collected by retro orbital route under mild anesthesia and serum was separated by centrifugation at 2000 rpm for 10 min and stored at -80°C for further use. TNF-α, IL-1β, and IL-10 were measured by using ELISA kits from R&D systems as per the instructions are given by the manufacturer, each sample was performed in duplicate and results were expressed in Pg/mL. The levels of hepatobiliary enzymes like AST, ALT, ALP, LDH, and bilirubin were estimated by commercially available assay kits from Accurex, India.

#### Measurement of Liver Antioxidant Enzymes and Lipid Peroxidation

After blood collection animals from all the groups were sacrificed using an overdose of ether anesthesia and liver was collected immediately and divided into two parts. One part was stored in 10% buffered formaldehyde for histopathology analysis and the second part was frozen in liquid nitrogen for biochemical estimation. The liver homogenate was prepared with 50 mM cold potassium phosphate buffer (pH 7.4) and centrifuged at 3000 rpm for 15 min (Jain et al., 2008). Supernatant was collected for the estimation of SOD, CAT using assay kits from Cayman, USA and TBA reacting substances (TBARS; Fan et al., 2009; Xia et al., 2013). All the experiments were conducted at 4°C.

#### Histopathology Examination of Liver

Livers were stored in 10% buffered formaldehyde and preserved for at least 24 h, dehydrated gradually with ethanol (70–100%), cleared in xylene and embedded in paraffin. Sections of 5 μm were prepared and stained with hematoxylin and eosin to examine under the microscope (10×) for histopathological changes (Bhardwaj et al., 2016; Choudhury A.J. et al., 2016).

### Effect of *Z. jujuba* Fractions against Carrageenan Induced Paw Edema

Adult male Wistar rats (*N* = 36), weighing 200–220 g were randomly divided into six groups (*n* = 6). Inflammation was induced in overnight fasted animals by intraplantar injection of 1% carrageenan (0.05 mL) to right hind paw (Arumugam et al., 2008). The following drug treatments were given to the animals before injecting the carrageenan.

**Group I:** Control animals (0.3% CMC orally)
**Group II:** Treatment with 10 mg/kg indomethacin orally.
**Groups III and IV:** Treatment with ZJME 200 mg/kg and 400 mg/kg orally.
**Groups V and VI:** Treatment with ZJWF 50 mg/kg and 100 mg/kg orally.

Water plethysmometer (Harvard apparatus, Panlab, Spain) was used to measure the paw volume at different time intervals (0, 1, 3, and 5 h) after the induction of inflammation. At the end of the experiment, blood was collected through retro orbital route from all the animals under mild anesthesia to measure the serum inflammatory markers. All the animals were sacrificed at the end of the experiment.

#### Measurement of Inflammatory Cytokine Levels

Serum was separated from blood by centrifugation at 2000 rpm for 10 min. Inflammatory cytokines like TNF-α and Il-1β were measured using ELISA kits from R&D systems, the USA as per the protocol is given by the manufacturer. All the treatments were done at 4°C and results were presented in pg/mL (Kandimalla et al., 2016c).

### Statistical Analysis

All the results were expressed as mean ± SD. One way ANOVA followed by Tukey’s multiple comparison tests were used to compare the different parameters between the groups. A *p*-value < 0.05 was considered as significant.

### Phytochemical Investigation of ZJME and ZJWF

Both ZJME and ZJWF were screened for phytochemicals to determine the nature of the chemical constituents present using following methods-

***Test for alkaloids:*** 0.5 g of the test sample was stirred with 5 ml of aqueous HCl acid (1%) on the water bath at 95°C. The reaction mixture was filtered using Whatman filter paper and few drops of dragendorff’s reagent were added to 1 ml of the filtrate. Formation of orange red precipitate was considered for the presence of alkaloids (Adegoke et al., 2010).
***Test for flavonoids:*** To 0.5 g of the test sample 5 ml of ethyl acetate was added and heated in a water bath and the reaction mixture was filtered. To 1 ml of the filtrate, few drops of ammonia solution were added and shaken vigorously. Formation of yellow color indicates the presence of flavonoids (Kalita et al., 2011).
***Test for phenolic compounds:*** About 50 mg of the test sample was dissolved in 5 ml of distilled water. To this mixture, 5% ferric chloride solution was added. Formation of dark green color indicated the presence of phenolic compounds (Kalita et al., 2011).
***Test for saponins:*** To a pinch of test sample 2–3 ml of distilled water was added and shake vigorously. Formation of foam indicated the presence of saponins (Adegoke et al., 2010; Kalita et al., 2011).
***Test for steroids:*** To 0.5 g of test sample 2 ml of acetic anhydride and 2 ml concentrated sulfuric acid was added. Change of color from violet to blue indicates the presence of steroids (Boxi et al., 2010; Kalita et al., 2011).
***Test for terpenes:*** To 0.5 g of test sample 3 ml of concentrated sulfuric acid and 2 ml of chloroform was added. Formation of reddish brown ring confirms the presence of terpenes (Obianime and Uche, 2008).
***Test for cardiac glycosides:*** Keller–Killani test was performed to determine the presence of cardiac glycosides. To 0.5 g of test sample, 2 ml of glacial acetic acid and one drop of ferric chloride was added. The reaction mixture was underlaid with 1 ml of concentrated sulphuric acid. Formation of the brown ring at the interface indicates the presence of cardiac glycosides.
***Test for oils and fats****:* A small amount of test sample was pressed between the two filter papers. Formation of oil stain indicates the presence of fats and oils (Kalita et al., 2011).
***Test for proteins and amino acids:*** 50 mg of the test sample was mixed with few ml of diluted HCl and filtered. To the filtrate, few drops of ninhydrin solution (10 mg of ninhydrin in 200 ml of Acetone) was added. Formation of purple color indicates the presence of proteins and amino acids.

### Total Phenolic and Flavonoid Content of ZJME and ZJWF

The total phenolic content of the ZJME and ZJWF were determined using the Folin–Ciocalteu assay. The results are expressed as grams of gallic acid equivalents per 100 g of dry extract. The total flavonoid content was determined by a colorimetric assay using rutin as a standard. The results are expressed as grams of rutin equivalents per 100 g of dry extract (Kandimalla et al., 2016d).

## Results and Discussion

### Extraction and Fractionation

Maceration of ZJRB (10 kg) with methanol yields 524 g of ZJME. Further, fractionation of ZJME (400 g) with different solvents of increasing polarity yields as follows- ZJHF (38 g), ZJCF (74 g), ZJEAF (27 g), and ZJWF (124 g). After the successful fractionation with all the solvents, the left out residue was also collected ZJMR (116 g). During the fractionation process, some amount of ZJME was lost (21 g).

### *In vitro* Antioxidant Activity of *Z. jujuba* Fractions

#### DPPH Radical Scavenging Activity

**FIGURE 2:**
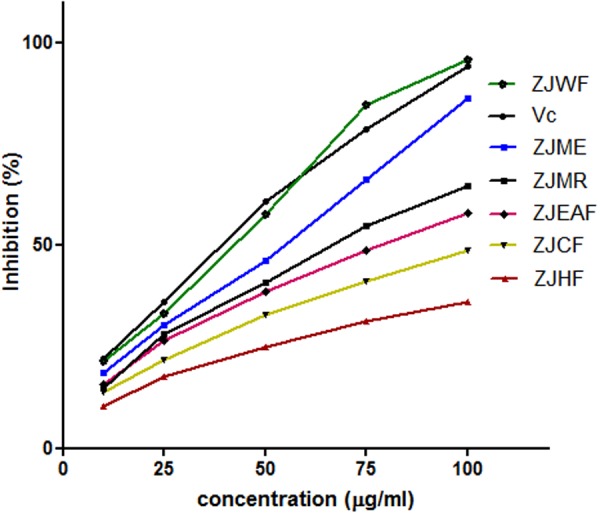
**DPPH radical scavenging activity of different fractions of *Z. jujuba* root bark.** ZJME, *Z. jujuba* root bark methanol extract; ZJHF, *Z. jujuba* root bark hexane fraction; ZJCF, *Z. jujuba* root bark chloroform fraction; ZJEAF, *Z. jujuba* root bark ethyl acetate fraction; ZJWF, *Z. jujuba* root bark water fraction; ZJMR, *Z. jujuba* root bark methanol residue; Vc, Ascorbic acid. All the results were expressed in mean ± SD (*n* = 3).

2,2-diphenyl-1-picryl hydrazyl is a free radical; the ability of test substance to neutralize the DPPH radical is considered as its anti-oxidant ability which is a widely accepted method to determine the *in vitro* antioxidant capacity (Liu et al., 2013). The free radical scavenging ability of *Z. jujuba* methanol extract (ZJME) and its fractions was given in **Figure 2**. Among all the fractions tested *Z. jujuba* water fraction (ZJWF) and ZJME showed potent response, which is comparable with standard ascorbic acid. At higher concentration, ZJWF showed potent activity than standard ascorbic acid. *Z. jujuba* hexane fraction (ZJHF), *Z. jujuba* chloroform fraction (ZJCF), *Z. jujuba* ethyl acetate fraction (ZJEAF) and *Z. jujuba* methanol residue (ZJMR) also exhibited dose dependent antioxidant activity but not up to the activity of ZJWF and ZJME. This result of this experiment suggests that both ZJWF and ZJME have the strong antioxidant ability.

#### Reducing Power Assay

**FIGURE 3:**
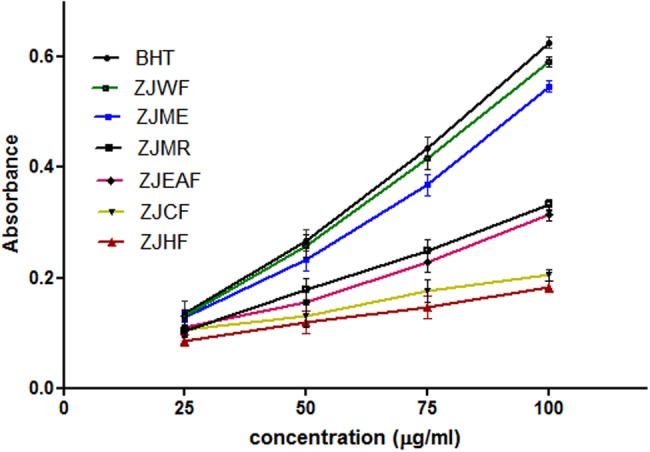
**Reducing power ability of different fractions of *Z. jujuba* root bark.** ZJME, *Z. jujuba* root bark methanol extract; ZJHF, *Z. jujuba* root bark hexane fraction; ZJCF, *Z. jujuba* root bark chloroform fraction; ZJEAF, *Z. jujuba* root bark ethyl acetate fraction; ZJWF, *Z. jujuba* root bark water fraction; ZJMR, *Z. jujuba* root bark methanol residue; BHT, Butylated hydroxyl toluene. All the results were expressed in mean ± SD (*n* = 3).

The ability of a test substance that reduce the Fe^3+^ to Fe^2+^ is considered as reducing power ability which is directly related to its antioxidant capacity. Reducing power ability of the substance can be measured spectrophotometrically (Meir et al., 1995). ZJME and ZJWF showed strong antioxidant activity and the later one is potent among all the fractions and extract which is comparable with standard BHT (**Figure 3**). So we selected ZJME and ZJWF for further pharmacological analysis.

#### Total Antioxidant Activity

Total antioxidant capacity of a test substance was measured by photochemiluminescence method in the Photochem instrument. This reaction depends on antioxidant concentration which follows the Guldberg–Waage law. The reaction of radicals with antioxidant molecules forms a stable compound which can be measured spectrophotometrically (Hic and Balic, 2012). Total antioxidant activity of ZJME and ZJWF was 69.76 nmol trolax equivalents and 94.27 nmol ascorbic acid equivalents, respectively.

#### Cytotoxicity Effect of ZJME and ZJWF in HepG2 Cells

ZJME and ZJWF at 5, 25, 50,125, and 250 μg/ml did not show the significant effect on HepG2 cell growth but at the highest dose 500 μg/mL both exhibited growth inhibition. So, we selected 50, 100, and 200 μg/ml of ZJME and ZJWF for evaluation of protective effect against CCl_4_ intoxicated HepG2 cells.

#### Protective Effect of ZJME and ZJWF in CCl_4_ Induced Toxicity on HepG2 Cells

In this study, the hepatoprotective ability of ZJME and ZJWF have ascertained in CCl_4_ intoxicated HepG2 cell lines. In this type of studies, HepG2 cells have been used as a substitute to hepatocytes. CCl_4_ is a well know toxin that induces lipid peroxidation in HepG2 cells and cause severe damage, which regarded as a convenient method to screen the medicinal plants extract and isolates against hepatotoxicity (Krithika et al., 2013). **Table 1**. shows the results of cell viability and LDH levels of HepG2 cells. Treatment with CCl_4_ significantly reduced the HepG2 cell viability and increased the LDH leakage into the culture media. ZJME and ZJWF treatment along with CCl_4_ reduce the toxic effect of CCl_4_ which was confirmed by increased cell viability and decreased LDH leakage. Results of this experiment demonstrated the hepatoprotective ability of ZJME and ZJWF against CCl_4_ intoxication in HepG2 cells; this might be due to the membrane stabilization. ZJWF showed potent response than ZJME, which explains that polar chemical components of ZJRB responsible for hepatoprotection. For further confirmation, we performed *in vivo* experiments.

**Table 1:** Effect of ZJME and ZJWF against CCl_4_ intoxicated HepG2 cell lines.

Group No.	Treatment		% Cell viability	LDH (U/L)
1	Group-I	Normal control	99.42 ± 0.26	96.64 ± 1.67
		DMSO control (0.25 % V/V)	98.19 ± 0.18	95.47 ± 1.45
		Silymarin control (200 μg/mL)	98.56 ± 0.21	97.18 ± 1.58
		ZJME (200 μg/mL) control	98.42 ± 0.23	96.44 ± 1.51
		ZJWF (200 μg/mL) control	98.17 ± 0.20	95.92 ± 1.68
2	Group-II	Toxin CCl4 control (1% v/v)	19.72 ± 0.26ˆ	197.64 ± 3.38ˆ
3	Group-III	Silymarin 50 μg/mL + CCl_4_ (1% v/v)	68.22 ± 0.21^∗^	123.37 ± 2.19^∗^
		Silymarin 100 μg/mL + CCl_4_ (1% v/v)	76.47 ± 0.18^∗^	115.42 ± 2.35^∗^
		Silymarin 200 μg/mL + CCl_4_ (1% v/v)	88.39 ± 0.24^∗^	106.42 ± 1.84^∗^
4	Group-IV	ZJME 50 μg/mL + CCl_4_ (1% v/v)	41.26 ± 1.42^∗^	176.54 ± 3.14^∗^
		ZJME 100 μg/mL + CCl_4_ (1% v/v)	49.53 ± 1.76^∗^	152.46 ± 2.54^∗^
		ZJME 200 μg/mL + CCl_4_ (1% v/v)	57.44 ± 1.62^∗^	135.27 ± 1.92^∗^
5	Group-V	ZJWF 50 μg/mL + CCl_4_ (1% v/v)	54.65 ± 2.16^∗^	146.58 ± 1.74^∗^
		ZJWF 100 μg/mL + CCl_4_ (1% v/v)	63.44 ± 1.85^∗^	130.64 ± 1.68^∗^
		ZJWF 200 μg/mL + CCl_4_ (1% v/v)	71.82 ± 1.42^∗^	119.61 ± 1.84^∗^


*All the results were expressed in mean ± S.D (*n* = 3). ˆ*P* < 0.05 in comparison of group-II with group-I. ^∗^*P* < 0.05 in comparison of group-III, IV, and V with group-II. ZJME, *Z. jujuba* root bark methanol extract; ZJWF, *Z. jujuba* root bark water fraction.*

#### Acute Toxicity Studies

After single large dose (2000 mg/kg) administration of ZJME and ZJWF, we observed the animals for toxic effects and behavioral changes. Both ZJME and ZJWF did not alter the heart rate, body temperature, respiratory rate, locomotor activity, salivation, corneal reflex, grip strength, abdominal tone, body tone, piloerection, tail elevation, tremors, convulsions, and twitches in animals. After 14 days of the observation period, no mortality was observed in the animals. The results of this study explain that both ZJME and ZJWF non-toxic to animals and we further continued for activity evaluation.

### Effect of ZJME and ZJWF against CCl_4_ Induced Hepatic Damage

#### Effect on Serum Hepatobiliary Enzyme Levels

**Table 2** shows the effect of ZJME and ZJWF on serum hepatobiliary enzymes like AST, ALT, ALP, and LDH levels. CCl_4_ treatment significantly raised all these serum biochemical markers. The 14 days of pretreatment with ZJME, ZJWF, and standard drug silymarin protects the liver from CCl_4_, which clearly observed by restored levels of all these markers. CCl_4_ is a well-known hepatotoxin that damages the plasma membrane of liver cells and causes necrosis. In deep inside the living system CCl_4_ converts into trichloromethyl radical (CCl_3_.), an active metabolite which covalently bonds with sulfhydryl groups in protein thiols of hepatocytes and causes lipid peroxidation and necrosis. During this necrotic stage, hepatobiliary enzymes release into blood circulation which is the reason behind the high enzyme levels after CCl_4_ treatment (Vuda et al., 2012). Results of this study demonstrated the hepatoprotective ability of both ZJME and ZJWF, where the latter one is more potent than former which states that polar chemical components of *Z. jujuba* were responsible for this activity.

**Table 2:** Effect of ZJME and ZJWF on serum and liver enzyme levels in CCl_4_ intoxicated rats.

Group No.	Group	Serum levels of				Liver levels of		
			
		AST (IU/l)	ALT (IU/l)	ALP (IU/l)	LDH (U/l)	SOD (U/mL)	CAT (nmol/min/mL)	TBARS (nmol/g tissue)
1	Control	41.6 ± 2.8	38.3 ± 2.4	94.5 ± 3.2	436.8 ± 15.4	9.8 ± 1.1	5.2 ± 0.8	158.5 ± 8.7
2	CCl_4_ treatment (1.5 mL/kg)	124.2 ± 6.1^$$$^	96.7 ± 5.8^$$$^	197.5 ± 9.7^$$$^	1022.9 ± 29.2^$$$^	1.9 ± 0.4^$$$^	0.8 ± 0.1^$$$^	342.8 ± 12.6 ^$$$^
3	Silymarin (100 mg/kg) + CCl_4_ (1.5 mL/kg)	52.8 ± 4.4^∗∗∗^	44.6 ± 3.5^∗∗∗^	108.2 ± 7.8^∗∗∗^	471.4 ± 20.6^∗∗∗^	7.7 ± 0.8^∗∗∗^	4.6 ± 0.5^∗∗∗^	171.8 ± 9.4^∗∗∗^
4	ZJME 200 mg/kg + CCl_4_ (1.5 mL/kg)	68.4 ± 4.7^∗∗∗^	57.4 ± 3.2^∗∗∗^	127.6 ± 6.6^∗∗∗^	546.2 ± 16.8^∗∗∗^	3.9 ± 0.8^∗∗∗^	3.4 ± 0.6^∗∗^	204.8 ± 9.2^∗∗∗^
5	ZJME 400 mg/kg + CCl_4_ (1.5 mL/kg)	60.1 ± 4.2^∗∗∗^	52.6 ± 2.9^∗∗∗^	120.4 ± 7.1^∗∗∗^	503.8 ± 17.3^∗∗∗^	5.5 ± 0.9^∗∗∗^	3.8 ± 0.4^∗∗∗^	189.6 ± 7.4^∗∗∗^
6	ZJWF 50 mg/kg + CCl_4_ (1.5 mL/kg)	63.7 ± 4.6^∗∗∗^	50.4 ± 3.3^∗∗∗^	124.8 ± 7.8^∗∗∗^	536.8 ± 17.6^∗∗∗^	5.3 ± 1.1^∗∗∗^	3.8 ± 0.6^∗∗∗^	190.8 ± 8.4^∗∗∗^
7	ZJWF 100 mg/kg + CCl_4_ (1.5 mL/kg)	55.2 ± 4.1^∗∗∗^	46.8 ± 2.8^∗∗∗^	115.6 ± 6.9^∗∗∗^	487.6 ± 15.2^∗∗∗^	7.0 ± 1.2^∗∗∗^	4.3 ± 0.5^∗∗∗^	175.1 ± 8.1^∗∗∗^


*All the results were expressed in mean ± S.D (*n* = 6). ^$$$^*P* < 0.001 in comparison of CCl_4_ alone treated animals with normal animals. ^∗∗∗^*P* < 0.001 and ^∗∗^*P* < 0.01 in comparison of drug treated animals with CCl_4_ alone treated animals. ZJME, *Z. jujuba* root bark methanol extract; ZJWF, *Z. jujuba* root bark water fraction.*

#### Effect on Lipid Peroxidation and Liver Antioxidant Enzymes

Superoxide dismutase and catalase are two powerful antioxidant enzymes that produce inside the body. SOD decomposes the superoxide radical (O_2_⋅^-^) into hydrogen peroxide (H_2_O_2_), which subsequently removed to water by CAT in the peroxisomes. CCl_4_ induction causes the significant decrease in antioxidant enzymes like SOD and CAT which leads to the lipid peroxidation and toxicity (Cheng et al., 2013). Treatment with CCl_4_ caused significantly (*P* < 0.001) increased the lipid peroxidation and declined SOD and CAT enzymes levels (**Table 2**). Treatment with ZJME, ZJWF, and standard drug silymarin showed significant protection from CCl_4_ which was demonstrated by reduced lipid peroxidation and high levels of antioxidant enzymes. ZJWF showed potent activity than ZJME which refers that the polar chemical components of ZJRB were responsible for this activity.

#### Effect on Serum Cytokine Levels

**FIGURE 4:**
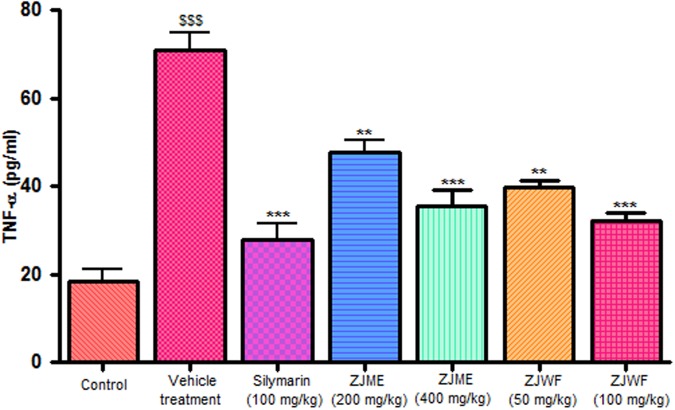
**Effect of different drug treatment on serum TNF-α levels.** All the results were expressed in mean ± SD (*n* = 6). ^$$$^*P* < 0.001 in comparison of CCl_4_ alone treated animals with normal animals. ^∗∗∗^*P* < 0.001 and ^∗∗^*P* < 0.01 in comparison of drug treated animals with CCl_4_ alone treated animals. ZJME, *Z. jujuba* root bark methanol extract; ZJWF, *Z. jujuba* root bark water fraction.

**FIGURE 5:**
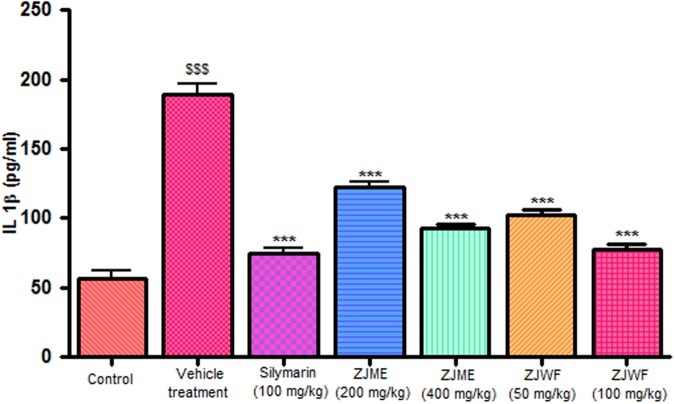
**Effect of different drug treatment on serum IL-1β levels.** All the results were expressed in mean ± SD (*n* = 6). ^$$$^*P* < 0.001 in comparison of CCl_4_ alone treated animals with normal animals. ^∗∗∗^*P* < 0.001 in comparison of drug treated animals with CCl_4_ alone treated animals. ZJME, *Z. jujuba* root bark methanol extract; ZJWF, *Z. jujuba* root bark water fraction.

**FIGURE 6:**
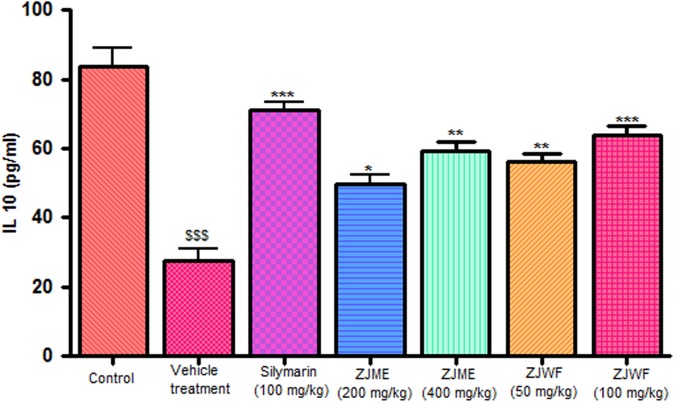
**Effect of different drug treatment on serum IL-10 levels.** All the results were expressed in mean ± SD (*n* = 6). ^$$$^*P* < 0.001 in comparison of CCl_4_ alone treated animals with normal animals. ^∗∗∗^*P* < 0.001, ^∗∗^*P* < 0.01, and ^∗^*P* < 0.05 in comparison of drug treated animals with CCl_4_ alone treated animals. ZJME, *Z. jujuba* root bark methanol extract; ZJWF, *Z. jujuba* root bark water fraction.

CCl_4_ metabolism triggers the liver kupffer cells, which leads to activation of inflammatory cytokines like TNF-α and IL-1β and reduces the anti-inflammatory cytokine IL-10. IL-1β is a powerful inflammatory cytokine, stimulates the macrophages and involves in prostaglandins production and neutrophil infiltration. Activation of this inflammatory cytokine cascade cause significant damage to the hepatocytes (Edwards et al., 1993; Ishiyama et al., 1995). CCl_4_ injection significantly raised the TNF-α, Il-1β, and reduced the IL-10 levels in the rats. ZJME and ZJWF treatment dose dependently reduced the TNF-α (**Figure 4**), IL-1β (**Figure 5**) production and increased the IL-10 levels (**Figure 6**) in animals intoxicated with CCl_4_. ZJWF was found to be more potent than ZJME which indicated that the active components are polar in nature.

#### Effect on Histopathology of Rat Liver

**FIGURE 7:**
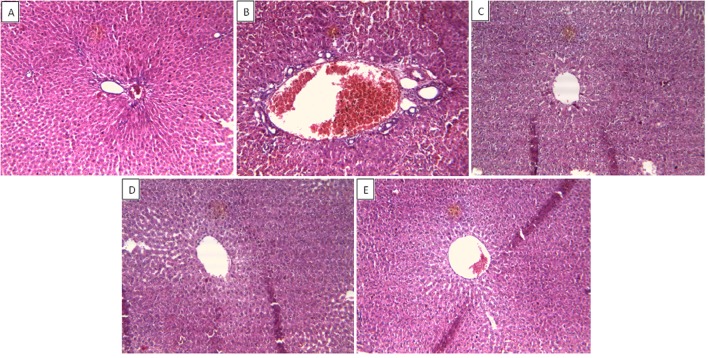
**Effect of drug treatment on liver histopathology of CCl_4_ intoxicated rats.** **(A)** Liver of control rat with no CCl_4_ treatment showing normal hepatocytes with no inflammatory cell infiltrate; ×10. **(B)** Liver of rat treated with CCl4 showing formation of septa and bridging necrosis with chronic inflammatory cells; ×10. **(C)** Liver of rat treated with CCl4 and 100 mg/kg silymarin showing normal hepatocytes with no inflammation and necrosis; ×10. **(D)** Liver of rat treated with CCl4 and 400 mg/kg of ZJME showing mild periportal inflammation and no necrosis; ×10. **(E)** Liver of rat treated with CCl4 and 100 mg/kg ZJWF showing absence of inflammatory cells or necrosis; ×10. ZJME, *Z. jujuba* root bark methanol extract; ZJWF, *Z. jujuba* root bark water fraction.

In support of the hepatoprotective response of ZJME and ZJWF, liver tissues from different treatment groups undergone for histopathology analysis. Hepatocytes with normal texture were observed with no cell infiltrate in control animals (**Figure 7A**). CCl_4_ intoxication causes demolishment of hepatocytes which was evidenced by the formation of bridging necrosis, collagen accumulation, large septa, and chronic inflammation (**Figure 7B**). Standard drug silymarin, ZJME (400 mg/kg) and ZJWF (100 mg/kg) pretreatment to the animals showed significant protection from CCl_4_, where normal liver architecture was observed with no necrosis and mild inflammation (**Figures 7C–E**). This histopathology data confirms the hepatoprotective ability of ZJRB and its polar chemical components were responsible for the activity.

### Effect of ZJME and ZJWF on Carrageenan Induced Paw Edema

#### Effect on Paw Volume

**FIGURE 8:**
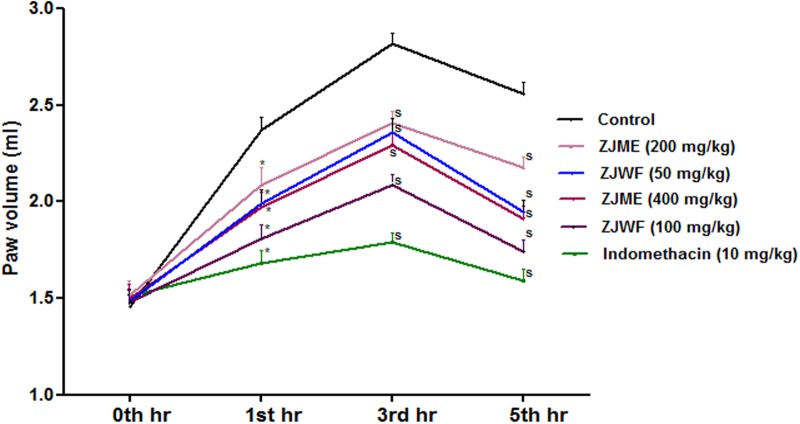
**Effect of *Z. jujuba* root bark fractions on carrageenan induced paw edema over 5 h.** All the results were expressed in mean ± SD (*n* = 6). S is *p* < 0.05 in comparison of drug treated animals with saline treated animals. ZJME, *Z. jujuba* root bark methanol extract; ZJWF, *Z. jujuba* root bark water fraction.

Inflammation occurs in two step process, in first step histamine and serotonin releases whereas the second step involves the secretion of prostaglandins and lysosomal bodies, which are the primary target for anti-inflammatory drugs in the market. Carrageenan induced inflammation in experimental animals is the widely accepted method to test the orally injected anti-inflammatory drugs (Marroquin-Segura et al., 2009). ZJME, ZJWF, and standard drug indomethacin reduced the paw volume significantly after carrageenan injection compared to non-treated animals (**Figure 8**). ZJWF showed potent response than ZJME which clearly indicates that polar chemical components of the ZJRB are responsible for this pharmacological response.

#### Effect on Serum Cytokine Levels

**FIGURE 9:**
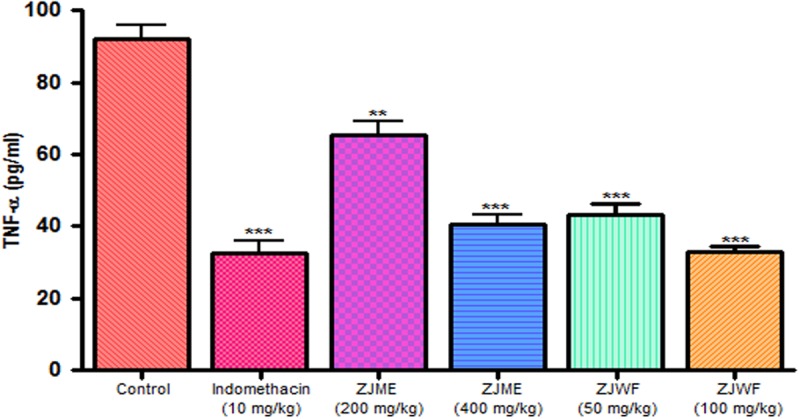
**Effect of different drug treatment on serum TNF-α levels of carrageenan induced paw edema rats.** All the results were expressed in mean ± SD (*n* = 6). ^∗∗∗^*P* < 0.001 and ^∗∗^*P* < 0.01 in comparison of drug treated animals with saline treated animals. ZJME, *Z. jujuba* root bark methanol extract; ZJWF, *Z. jujuba* root bark water fraction.

**FIGURE 10:**
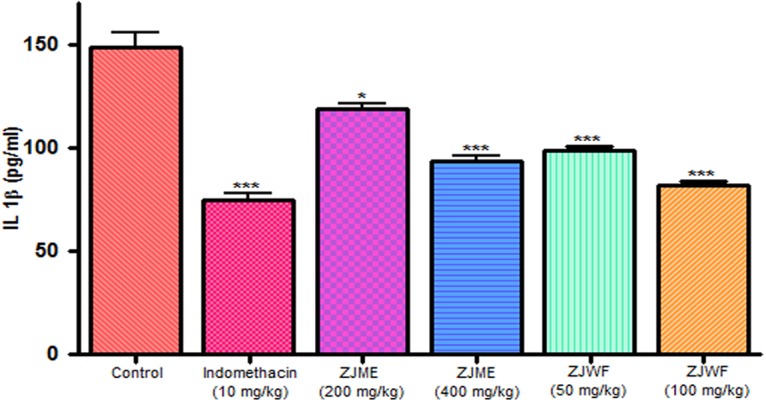
**Effect of different drug treatment on serum IL-1β levels of carrageenan induced paw edema rats.** All the results were expressed in mean ± SD (*n* = 6). ^∗∗∗^*P* < 0.001 and ^∗^*P* < 0.05 in comparison of drug treated animals with saline treated animals. ZJME, *Z. jujuba* root bark methanol extract; ZJWF, *Z. jujuba* root bark water fraction.

IL-1β and TNF-α are two powerful inflammatory cytokines which play a major role in the activation of the prostaglandin and macrophages. Intraplantar injection of carrageenan to rats causes the significant rise in TNF-α and IL-1β levels. Pretreatment of indomethacin, ZJME, and ZJWF before carrageenan shot inhibits the cytokines levels (**Figures 9** and **10**) compared to non-treated animals. ZJWF showed potent response than ZJME which demonstrates that anti-inflammatory compounds of ZJRB were polar in nature.

### Phytochemical Investigation of ZJME and ZJWF

The pharmacologically active fractions ZJME and ZJWF were subjected to qualitative phytochemical analysis to determine the nature of the active compounds. ZJME showed the presence of alkaloids, flavonoids, phenolic compounds, terpenes, saponins, and steroids where alkaloids and steroids were absent in ZJWF (**Table 3**). Based on these findings further investigation of bioactive compounds isolation is in progress in our laboratory and will be reported in our further communications.

**Table 3:** Phytochemical constituents of ZJME and ZJWF.

S.No	Phytochemicals	ZJME	ZJWF
1	Alkaloids	+	–
2	Flavonoids	+	+
3	Phenolic compounds	+	+
4	Steroids	+	–
5	Terpenes	+	+
6	Saponins	+	+
7	Cardiac glycosides	–	–
8	Oils and fats	–	–
9	Proteins and amino acids	–	–


*ZJME, *Z. jujuba* root bark methanol extract; ZJWF, *Z. jujuba* root bark water fraction. + Present; – absent.*

### Total Phenolic and Flavonoid Content of ZJME and ZJWF

Antioxidant capacity of a plant fraction or extract correlates with the presence of the phenolic type of compounds (Bravo, 1998; Huang et al., 2010; Chen et al., 2011). Total phenolic content of ZJME and ZJWF was found to be 246.4 ± 4.37 mg and 322.6 ± 3.72 mg gallic acid equivalents/g extract, respectively. The total flavonoid content of ZJME and ZJWF was found to be 4.9 ± 0.42 mg and 6.2 ± 0.46 mg rutin equivalents/g extract, respectively. Results of this study explain that ZJWF contains rich amount of phenolic and flavonoid compounds than ZJME. Further investigation on enrichment and isolation of bioactive compounds is ongoing in our laboratory.

## Conclusion

This study demonstrated the protective ability of ZJRB against hepatotoxicity and inflammation. Hepatoprotective activity may be attributing to its strong antioxidant ability which reduced the oxidative stress and lipid peroxidation. Further, ZJRB showed anti-inflammatory activity through inhibiting the pro-inflammatory cytokines. Water fraction of methanol extract showed potent response toward all the *in vitro* and *in vivo* tests conducted, this may be due to polar chemical components of the plant. Therefore the study scientifically validates the traditional usage of ZJRB for the treatment of liver ailments and inflammation. Successful isolation of the biomarker can potentially contribute toward the development of new drug entity.

## Author Contributions

RK designed the whole study and performed all the *in vitro* and *in vivo* experiments. SK, BC, SM, and BK helped RK in different phases of the study. SD contributed towards study design, supervision and compiling the results. KK performed and analyzed the histopathology of liver tissue. RD helped in literature survey and study design. JK contributed towards the study design, manuscript correction, results compiling, and supervision of the whole study.

## Conflict of Interest Statement

The authors declare that the research was conducted in the absence of any commercial or financial relationships that could be construed as a potential conflict of interest.

The results of control, toxin, and standard treated groups have been used in another study of ours (DOI: 10.3389/fphar.2016.00168).
